# Long valley lifetime of dark excitons in single-layer WSe_2_

**DOI:** 10.1038/s41467-019-12129-1

**Published:** 2019-09-06

**Authors:** Yanhao Tang, Kin Fai Mak, Jie Shan

**Affiliations:** 1 School of Applied and Engineering Physics, Cornell University, Ithaca, NY USA; 2 Laboratory of Atomic and Solid State Physics, Cornell University, Ithaca, NY USA; 3 Kavli Institute at Cornell for Nanoscale Science, Ithaca, NY USA

**Keywords:** Two-dimensional materials, Spintronics, Electronic properties and materials

## Abstract

Single-layer transition metal dichalcogenides provide a promising material system to explore the electron’s valley degree of freedom as a quantum information carrier. The valley degree of freedom can be directly accessed by means of optical excitation. However, rapid valley relaxation of optically excited electron-hole pairs (excitons) through the exchange interaction has been a major roadblock. Theoretically such valley relaxation is suppressed in dark excitons, suggesting a potential route for long valley lifetimes. Here we develop a waveguide-based method to detect time-resolved and energy-resolved dark exciton emission in single-layer WSe_2_, which involves spin-forbidden optical transitions with an out-of-plane dipole moment. The valley degree of freedom of dark excitons is accessed through the valley-dependent Zeeman effect under an out-of-plane magnetic field. We find a short valley lifetime for the dark neutral exciton, likely due to the short-range electron-hole exchange, but long valley lifetimes exceeding several nanoseconds for the dark charged excitons.

## Abstract (web summary)

The valley degree of freedom in monolayer transition metal dichalcogenides can be addressed by optical means. Here, the authors develop a waveguide-based method to detect emission from dark excitons in single-layer WSe2, access the valley degree of freedom through the Zeeman effect, and demonstrate long valley lifetime of charged dark excitons.

## Introduction

Single-layer transition metal dichalcogenides (TMDs, MX_2_: M = Mo, W; X = S, Se) are direct band-gap semiconductors with direct gaps located at the K and K′ valleys of the Brillouin zone^1,2^. Both valence and conduction bands are spin-split at the two valleys by strong spin-orbit coupling. Excitons formed by Coulomb interactions from electrons and holes of antiparallel spins are “bright” (optically active), and from electrons and holes of parallel spins are “dark” (optically inactive). The bright excitons exhibit strong valley circular dichroism (i.e. each handedness of circularly polarized light couples only to one of the two valleys), which provides an effective means to access the valley degree of freedom^3–5^. Such valley circular dichroism has triggered intense interest in single-layer TMDs as potential candidates for valleytronic applications^6–8^, which desire a long valley lifetime. However, valley relaxation is very fast (order of 10 ps) for the bright neutral^9–11^ and charged excitons^12–14^. The fast valley relaxation is attributed to the long-range electron-hole exchange interaction^15,16^, which mixes the two valley exciton states. On the other hand, intervalley scattering of the dark excitons would require a spin flip, which does not occur through the long-range exchange interaction^15^. Long-lived valley-polarized dark excitons are thus possible. In tungsten-based TMDs the dark excitons have a lower energy than the bright excitons and have recently been shown long-lived^17,18^. Direct measurement of the valley lifetime of the dark excitons, however, remains challenging. The spin-forbidden excitons have an out-of-plane (OP) transition dipole moment^19–21^, making their detection difficult with conventional far-field optical techniques. In addition, unlike for the bright excitons, there are no valley-dependent optical selection rules for the dark excitons that can be utilized for direct optical access of the valley degree of freedom.

Here we study the valley dynamics of dark excitons in single-layer WSe_2_ by time-resolved photoluminescence (PL) spectroscopy and observe long valley lifetimes exceeding several nanoseconds for the dark charged excitons. The measurement has been enabled by coupling WSe_2_ to a GaSe waveguide using a conventional far-field setup. The approach complements the reported methods for the dark exciton detection^17,19,20,22,23^, for instance, by applying a large in-plane magnetic field^17,23^, and near-field coupling to surface-plasmon polaritons^19^ or an antenna-tip^22^. The waveguide-based method is highly efficient, unique in its selective detection of emission originated from both an OP and an in-plane (IP) transition dipole moment by polarization, and practical for device applications. We also show that the valley-polarized dark excitons can be initiated through scattering of the valley-polarized bright excitons. Under an OP magnetic field, the valley-polarized dark excitons from the K and K’ valleys can be further separated by the Zeeman shift, enabling the measurement of the valley polarization and dynamics. The long valley lifetimes revealed by our experiment for dark charged excitons have a distinct physical origin from that found in localized emitters^24^, resident carriers^25–29^ and interlayer excitons^30^.

## Results

### Resolving the IP and OP dipole emission

**Fig. 1:**
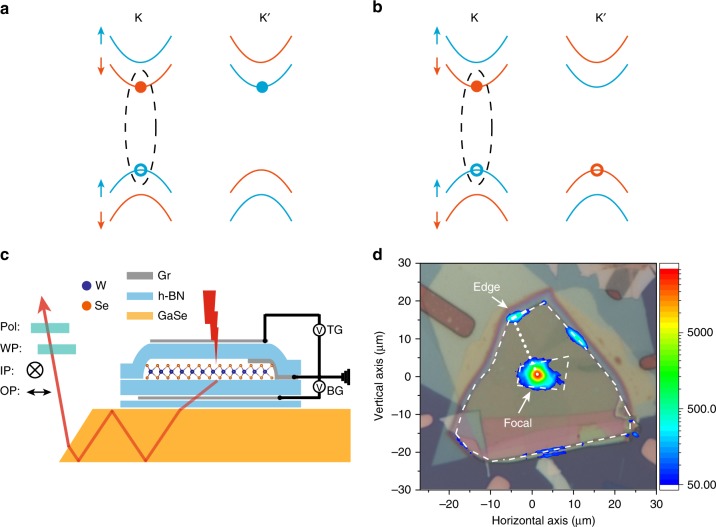
Dark excitons in WSe_2_ and experimental geometry. **a**, **b** Electronic configuration of a dark electron trion (**a**) and dark hole trion (**b**) in single-layer WSe_2_. Blue and orange curves represent electronic bands with electron spin up and spin down, respectively. The hole spin is opposite to what’s shown for the electron spin. Dashed ellipses indicate the electron-hole pairs involved in the recombination. **c** Schematic side view of a dual-gated WSe_2_ device on a GaSe waveguide. WSe_2_ is excited by a focused light beam (red lightning symbol) and the resultant PL guided by the waveguide (red arrowed line) is detected. The IP and OP emission dipoles are selected by a half-wave plate (WP) and a polarizer (pol). WSe_2_ is grounded. TG and BG are the top and bottom gate voltages, respectively. **d** Optical reflection and PL images (overlaid) of a typical device. Inner and outer white dashed lines show the boundary of WSe_2_ and GaSe, respectively. The dotted white line, which is perpendicular to the edge, is referred to as the focal-edge line. The color bar represents the PL intensity.

The schematic of the device geometry is shown in Fig. 1c. Single-layer WSe_2_ is contacted with a few-layer graphite electrode, and can be gated by a top and a bottom graphite gate with hexagonal boron nitride (h-BN) gate dielectrics. The WSe_2_ field-effect device is positioned on top of a GaSe layer of several-hundred-nanometer thickness, which functions as a slab waveguide^31^. Such a thickness is required to support at least one optical mode. A combination of a half-wave plate and a polarizer selects the emission from an IP or an OP dipole. The entire device is on a Si substrate with an oxide layer. GaSe was chosen for the waveguide since it has a relatively high optical refractive index (about 2.9), low loss in the WSe_2_ PL spectral range (several cm^−1^)^32^, and a van der Waals crystal structure that allows us to build the entire device using the van der Waals heterostructure platform. (See Methods for details on the device fabrication).

In Fig. 1d, an optical image of a typical device is overlaid with its PL image. The PL collected in the back-reflection geometry is observed not only from the focal point, at which the optical excitation is focused, but also from several edges of the waveguide. Light is easier to couple out of the waveguide at edge faces that are perpendicular to the wave propagation direction (the lines connecting the edge and the focal point are referred to as the focal-edge lines below). The output intensity varies at different locations and as large as 5% of the intensity from the focal point has been observed at individual edges. The value presumably can be improved by optimizing the waveguide such as the tilt angle of the exit face (Fig. 1c). In the current generation of devices we have used GaSe as exfoliated from bulk crystals. Below we present the results from one edge (indicated by an arrow in Fig. 1d). The results from other locations are similar. All measurements were performed at 5 K on single-layer WSe_2_ with varying doping densities while the electric field perpendicular to the layer was kept approximately at zero (The two symmetric gates were set to the same voltage). (See Methods for details on the PL measurements.)

**Fig. 2:**
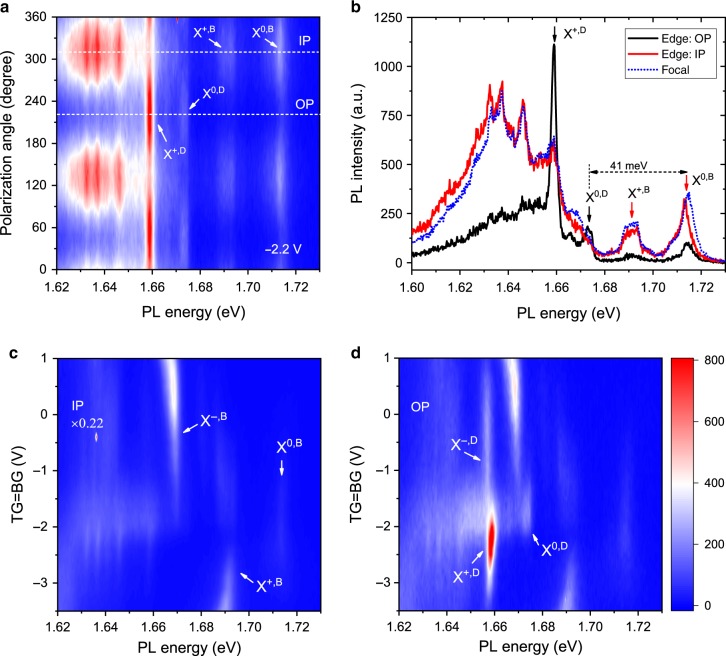
Resolving IP and OP dipoles by polarization. **a** Contour plot of the edge PL spectrum as a function of polarization direction for a hole-doped WSe_2_ sample (both gates at −2.2 V). Dashed lines indicate the polarization corresponding to the OP and IP channels. **b** Comparison of the PL spectrum from the edge OP (black line) and IP (red line) channels and from the focal point (blue dotted line). The latter is rescaled to match the edge IP channel spectrum. **c**, **d** Contour plot of the edge PL spectrum as a function of gate voltage for the IP (**c**) and OP (**d**) channels. The IP channel is rescaled by a factor of 0.22 so that the PL intensity of the bright electron trion X^−,B^ in two channels have a comparable intensity. The two gates are set to the same voltage, which varies only the doping density in WSe_2_ with symmetric top (TG) and bottom (BG) gates. The color bar represents the PL intensity in **a**, **c**, **d**. X^0,B^, X^+,B^, X^0,D^, X^+,D^ and X^−,D^ denote the bright exciton, bright hole trion, dark exciton, dark hole trion and dark electron trion, respectively. The energy splitting between the bright and dark exciton (41 meV) in **b** agrees with the literature value^19,20^.

Figure 2a shows the emission spectrum collected from the edge as a function of polarization direction (vertical axis). WSe_2_ is hole-doped in this example (gate voltages were −2.2 V). All sharp spectral features show a two-fold symmetry and can be divided into two groups with orthogonal polarizations. They exhibit maximum intensities when the polarizer transmission axis is set either perpendicular or parallel to the focal-edge line (labeled IP and OP, respectively, in Fig. 2a). The IP channel is dominated by two features, X^0,B^ and X^+,B^, at the high-energy end and multiple sharp features on a broad background at the low-energy end. They correspond to the bright neutral exciton, the bright positively charged exciton (*i.e*. hole trion), and the localized or finite-momentum bright excitons in single-layer WSe_2_^33–35^. The OP channel is dominated by two new features, X^0,D^ and X^+,D^, which are assigned as the dark neutral exciton and the dark hole trion (see Fig. 1a, b for electronic configurations), respectively, according to the literature^17,19^. Since the bright and dark excitons are known to be IP and OP dipoles^17,19,20,22,23,36^, this result shows that our device geometry can selectively detect the IP and OP dipole emission by polarization.

In Fig. 2b we compare the PL spectra collected from the edge and from the focal point. The IP channel is nearly identical to the (rescaled) spectrum from the focal point, further supporting that the IP channel is dominated by the IP dipole emission. The red shift of the bright exciton PL collected from the edge originates from re-absorption of the PL by WSe_2_ during its propagation in the waveguide. Figure 2c, d show the doping dependences of the PL from the two channels. Both positively and negatively charged excitons (bright or dark) can be accessed in a single device by electrostatic gating. The OP channel intensity is at least 20% of the IP channel intensity. The extinction of the IP dipole emission in the OP channel is not perfect (Fig. 2d), but it can be subtracted by using the scaled IP channel as background and has negligible impact on the analysis of the dark exciton dynamics below.

### Resolving the valley state of dark excitons

**Fig. 3:**
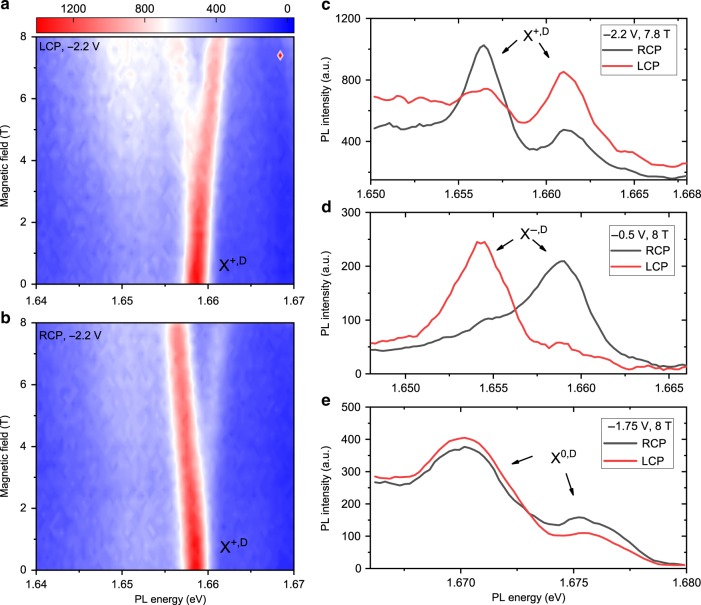
Resolving the valley degree of freedom of dark excitons by the Zeeman shift. **a**, **b** Contour plot of the PL spectrum of the OP channel as a function of magnetic field for a hole doped WSe_2_ sample (both gates at −2.2 V). **a** is for the LCP excitation and **b** for the RCP excitation. The color bar represents the PL intensity. **c**–**e** PL spectra of the OP channel under RCP (black line) and LCP (red line) excitation. The out-of-plane field is about 8 T. The gate voltages −2.2 V (**c**), −0.5 V (**d**), and −1.75 V (**e**) correspond to a hole-doped, electron-doped, and neutral sample, respectively.

To resolve the valley degree of freedom of the dark exciton, we lift the valley degeneracy by the Zeeman effect under an OP magnetic field. Figure 3a is the PL spectrum from the OP channel as a function of magnetic field (vertical axis). Single-layer WSe_2_ is hole doped (both gates at −2.2 V) and is excited by left-circularly-polarized (LCP) light at 1.96 eV (above the bright exciton fundamental resonance, but below the B exciton resonance). We observe that the PL of dark hole trions X^+,D^ splits into two peaks as the magnetic field increases, and the higher-energy peak is brighter than the lower-energy peak. The behavior of X^+,D^ is similar under right-circularly-polarized (RCP) excitation (Fig. 3b), but the brightness of the two peaks is reversed. Figure 3c shows a line cut of Fig. 3a, b at 7.8 T. The observed peak splitting corresponds to a g-factor of 10.9 ± 0.1, which is consistent with earlier studies of dark trions^17^. The fact that either of the Zeeman-split states can have a higher intensity than the other under LCP/RCP excitation demonstrates that the valley polarization can be optically initialized (through the bright exciton relaxation) and maintained in the dark hole trion. The microscopic scattering process of the bright to dark exciton states is not well understood and warrants future studies.

By making reference to the behavior of the bright hole trion under the same experiential conditions (for which the valley-dependent optical selection rules apply, see Supplementary Note 1), we determine that the higher- and lower-energy Zeeman-split states of X^+,D^ correspond to the K and K′ valleys, respectively. The valley contrast can be estimated as $\rho \approx \frac{{I_{{\mathrm{K}}\prime } \ - \ I_{\mathrm{K}}}}{{I_{{\mathrm{K}}\prime } \ + \ I_{\mathrm{K}}}}$, where *I*_K(K′)_ is the integrated PL intensity of the Zeeman-split K(K′) valley states. The PL intensity is a reasonable approximation of the valley population when the Zeeman splitting is insignificant. The steady-state value of *ρ* for the dark hole trion is found to be −0.44 ± 0.06 and 0.53 ± 0.05 for LCP and RCP excitation, respectively. The sign change of valley polarization supports that the valley polarization is due to the optical excitation. A similar steady-state valley contrast is estimated for the dark electron trion X^−,D^ (Fig. 3d). But there is no sign change of valley contrast for the dark neutral exciton X^0,D^ (Fig. 3e). The latter suggests rapid valley relaxation within the neutral dark exciton lifetime (about 100 ps^18^) and the observed valley polarization is mainly due to thermalization under a finite magnetic field. Below we focus only on the dynamics of dark hole trions (X^+,D^). (See Supplementary Note 1 for detailed analysis of the g-factor, the valley index for the Zeeman-split states, and the steady-state valley contrast of all types of dark excitons.)

### Valley dynamics of dark excitons

**Fig. 4:**
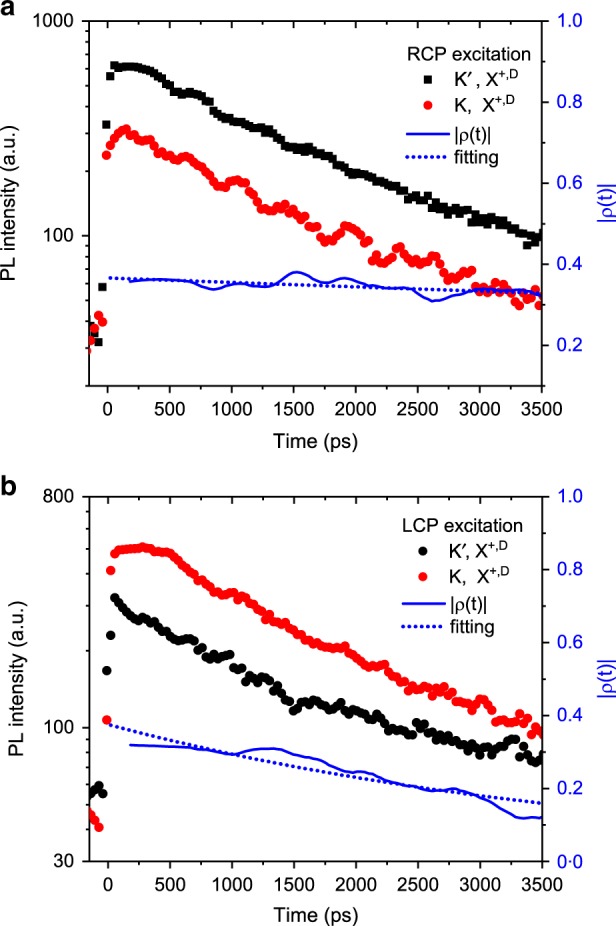
Dark exciton valley dynamics. Time-resolved PL of the dark hole trion in a hole doped WSe_2_ sample (both gates at −2.2 V) under a magnetic field of 8 T for the RCP (**a**) and LCP (**b**) excitation. Black and red symbols represent the PL of the Zeeman-split dark hole trion associated with the K′ and K valleys, respectively. The solid blue curves are the valley contrast $|\rho \left( t \right)|$ as defined in the main text. The dotted blue curves are a single-exponential fit, revealing a decay time constant of 32 ± 4 ns and 4.1 ± 0.2 ns, respectively, for the RCP and LCP excitation

We perform the energy-resolved time-correlated single-photon counting (TCSPC) measurements on doped WSe_2_ under an OP magnetic field of 8 T. The sample was excited by circularly polarized optical pulses of 180 fs in duration, 79 MHz in repetition rate, and peaked at 1.82 eV. PL was collected from the edge in the OP channel. The setup has a temporal resolution of 36 ps (the full-width-half-maximum of the instrument response function). (See Methods for details on the TCSPC measurements and Supplementary Note 2 for the PL dynamics of different types of excitons). Figure 4a shows the time-resolved PL at the peaks of X^+,D^ from the K and K′ valleys under RCP excitation. The traces have been deconvoluted with the instrument response function and filtered to remove high-frequency noise above 3 GHz, which arises mainly from the numerical deconvolution process. For the RCP excitation, the PL intensity of the K′ valley trion is about twice of the intensity of the K valley trion. For the LCP excitation, the intensity trend of the K and K′ valleys is reversed (Fig. 4b). This is consistent with the steady-state PL measurements (Fig. 3).

There is a small background before the arrival of the excitation pulse. This is likely the contribution of long-lived localized excitons (the broad background on which X^+,D^ sits in Fig. 2b) that are excited by previous optical pulses. To eliminate the influence of such background on our analysis of the valley lifetime, we have restricted our window of interest (3.5 ns) so that the count at any time is at least twice the background value. (See Supplementary Note 3 for more discussions.) We evaluate the valley contrast $|\rho \left( t \right)|$ using the PL intensities and obtain a decay time constant of 32 ± 4 ns and 4.1 ± 0.2 ns, respectively, for the RCP and LCP excitation from fitting to a single exponential function (dotted lines). The longer valley lifetime obtained for the RCP excitation is expected since the Zeeman effect breaks the degeneracy of the K and K′ states and scattering from the lower-energy state to the higher-energy state is suppressed. We therefore place the lower bound of the dark hole trion valley lifetime at ~4 ns. We note that the valley lifetime can also be measured by comparing the PL intensity at either the higher- or the lower-energy peak under LCP and RCP excitation. Similar results are obtained for the dark hole trions (Supplementary Note 4). Similarly, the lower bound of the valley lifetime of dark electron trions is found to be ~3.5 ns (Supplementary Note 5).

## Discussion

We have observed a long-lived few-nanosecond valley polarization for the dark charged excitons, but not for the dark neutral exciton. The initial theory has argued that the intervalley scattering of the dark neutral exciton that requires a spin flip could not occur through the long-range electron-hole exchange (the lowest-order exchange)^15^. However, more recent theoretical works have predicted that the short-range electron-hole exchange (a second-order exchange)^37,38^ could mix the two dark neutral exciton states at the K and K′ valleys to form two new states with a small splitting. The lower-energy dark neutral exciton is a truly dark state, which is both spin- and electric-dipole-forbidden. The higher-energy state is a nearly dark state, which is spin-forbidden, but dipole-allowed with an OP dipole. Recent magneto-luminescence experiments have reported a zero-field splitting of 0.6 meV between these states in single-layer WSe_2_^18^. In our experiment the observed dark neutral exciton is the spin-forbidden exciton with an OP dipole. The observed short valley lifetime could therefore be attributed to the short-range electron-hole exchange. On the other hand, the dark charged exciton is composed of a dark exciton in one valley and a hole (or an electron) in the other valley (Fig. 1a, b). The dark charged excitons are not prone to intervalley scattering through the electron-hole exchange because they have non-zero but opposite momentum at the two valleys.

In conclusion, we have developed a waveguide-based method for resolving the dipole orientation in layered materials, which is challenging with conventional far-field optical techniques. By integrating single-layer WSe_2_ dual-gate field-effect devices directly into the waveguide, we have been able to time-resolve and valley-resolve the emission from the dark exciton states under an OP magnetic field. We have determined a valley polarization lifetime exceeding several nanoseconds for the dark charged excitons, which may have implications for valley-based information storage and processing applications.

## Methods

### Device fabrication

The waveguide-coupled dual-gate WSe_2_ devices were built from exfoliated van der Waals materials using a layer-by-layer dry transfer method^39^. Atomically thin h-BN, graphite and WSe_2_ flakes were exfoliated from their bulk crystals onto silicon substrates, which were pretreated with ozone plasma. The thickness of single-layer WSe_2_ flakes was estimated from their optical contrast and confirmed by the PL spectra. The h-BN flakes of similar thickness were used as the gate dielectric for both the top and bottom gates. GaSe was exfoliated onto polydimethylsiloxane (PDMS) to obtain hundred-nm-thick slabs of large size for the waveguide. The stamp used for the transfer was made of polypropylene-carbonate-coated PDMS covered by a polycarbonate (PC) layer. In the transfer process, a GaSe slab was first picked up from PDMS and released onto a 300-nm-SiO_2_/Si chip with pre-patterned gold electrodes at around 180 °C. The residual of the stamp on GaSe was removed by submerging the whole chip in chloroform for a few minutes followed by a rinse in isopropyl alcohol. Other atomically thin flakes were assembled layer-by-layer first and the entire stack was then released onto the GaSe waveguide. The stamp residual was removed using the same procedure before the optical measurements.

### Photoluminescence measurements at low temperature

Devices were mounted in a close-cycle cryostat (a Montana or an Attocube system). For steady-state PL measurements, a continuous-wave (CW) laser at 633 nm with power less than 100 μW was used to excite WSe_2_. For time-resolved PL measurements, output from a Ti:sapphire oscillator (Coherent, Chameleon Ultra II) with a repetition rate of 79 MHz, a photon energy centered at 1.818 eV and an average power of 110 μW was used to excite WSe_2_. The excitation beam was focused to a beam radius of 1 μm on the WSe_2_ samples using a microscope objective of a numerical aperture of 0.6 or 0.8. The PL was collected by the same objective in the back-reflection geometry, and focused onto an entrance slit of a monochromator (Princeton Instruments, HRS-300MS) with a 600 grs mm^−1^ grating. One exit port of the monochromator is connected to a liquid-nitrogen cooled CCD camera (Princeton Instruments, PYL-400BRX) for steady-state PL measurements. The second exit port is coupled to a single-photon detector (SPD from Picoquant, PD-050-CTD) with a telescope which reduces the size of the image from the exit port to the SPD by a factor of two to increase the PL collection efficiency. The output of the SPD is registered in Picoharp300 for time-correlated single-photon counting (TCSPC) measurements. The setup has a temporal resolution of 36 ps, as determined from the full-width-half-maximum of the instrumental response function.

## Supplementary information

Supplementary Information

## Data Availability

The data supporting the plots within this paper and other findings of this study are available from the corresponding author upon reasonable request.
