# Supplementary material for: Long valley lifetime of dark excitons in single-layer WSe2
Source: Nat Commun. 2019 Sep 6;10:4047. doi: 10.1038/s41467-019-12129-1 (PMC6731252; doi:10.1038/s41467-019-12129-1)
Supplement: Supplementary file 1 — Supplementary Information [file 41467_2019_12129_MOESM1_ESM.pdf]

Supplementary Information for
Long valley lifetime of dark excitons in single-layer WSe_2

Tang et al.

Supplementary Note 1: Steady-state PL for dark excitons under a magnetic field

The photoluminescence (PL) spectrum of the out-of-plane (OP) channel was measured for both neutral and doped WSe_2 as a function of OP magnetic field. Both the left- and right-circularly-polarized (LCP and RCP) excitations above the bright exciton fundamental resonance have been used. We fit each PL spectrum using a superposition of two Gaussians and a background (Supplementary Fig. 1-3 for three different types of dark excitons). A substantial Zeeman splitting is observed for the dark hole trion, electron trion, and neutral exciton in Supplementary Fig. 4 – 6, respectively. The g-factor is determined to be 10.9$\pm$0.1, 10.2$\pm$0.3, and 10.8$\pm$0.5 accordingly. The g-factor of the neutral dark exciton is slightly higher than the previously reported values (9.16 by Metteo Barbone et al.[1], 9.4 by C. Robert et al.[2], and 9.75 by Li, Z. et al.[3]).

We also estimate the valley contrast $\rho \approx \frac{I_{\mathrm{K'}}-I_{\mathrm{K}}}{I_{\mathrm{K'}}+I_{\mathrm{K}}}$ from the integrated intensity of the two Gaussian peaks $I_{\mathrm{K(K')}}$. The valley contrast is evaluated to be 0.54$\pm$0.05 and -0.44$\pm$0.06 for the dark hole trion measured with the RCP and LCP excitation, respectively (Supplementary Fig. 1). A similar analysis has been carried out for the dark electron trion (Supplementary Fig. 2) and a valley contrast of 0.46$\pm$0.09 and -0.68$\pm$0.12 is obtained using the RCP and LCP excitation, respectively. In contrast, the valley contrast for dark neutral exciton (Supplementary Fig. 3) is -0.35$\pm$0.05 and -0.58$\pm$0.08, without any sign change, suggesting short valley lifetimes, and the valley polarization, due to thermalization of the valley states under an OP magnetic field. All errors of the valley contrast represent the fitting error.

In order to determine the valley state of the dark excitons, we measure the PL of the bright excitons through the in-plane (IP) channel under the same experimental conditions and rely on the valley optical selection rules that apply to the bright excitons. One example is shown in Supplementary Fig. 7 for an electron-doped WSe_2 sample (both gates at 0 V) under 8 T. We observe that the LCP and RCP excitations (that couple exclusively to the K and K’ valleys) address the *lower*- and *higher-energy* Zeeman-split states of the bright electron trion, respectively. This is the same order for $\mathrm{X}^{-,\mathrm{D}}$ and $\mathrm{X}^{0,\mathrm{D}}$ shown in Figure 3 of the main text. Accordingly, we assign the brighter Zeeman-split peak of the dark excitons with the LCP and RCP excitation to the K and K’ valleys, respectively. The order is reversed for $\mathrm{X}^{+,\mathrm{D}}$.

Supplementary Note 2: PL dynamics of excitons

The time-resolved PL at different energies of both the IP and OP channels has been measured under zero magnetic field using the time-correlated single-photon-counting method. Supplementary Supplementary Figure shows the time-resolved PL monitored at the peak energy of the bright exciton $\mathrm{X}^{0,\mathrm{B}}$ and the bright trion $\mathrm{X}^{+,\mathrm{B}}$ together with the instrument response function (IRF). They show a fast rise and a slow decay. The decay of the bright exciton and trion is only slightly slower than the IRF. The results from the OP and IP channels are also nearly identical except the PL intensity (not shown).

On the other hand, the dark trion shows a substantially longer decay time. Supplementary Supplementary Figure shows the time-resolved PL at the dark hole trion energy from the IP and OP channels. The traces have been deconvoluted with the measured IRF. The two channels differ significantly for the first 3.5 ns, with a much slower decay for the OP

channel, and become similar gradually afterwards. The latter can be attributed to the contribution of the localized excitons with very long population lifetimes. The localized excitons presumably do not have a well-defined emission dipole direction. The PL dynamics of OP channel can be well described by a three-exponential fit (see details in Supplementary Note 3). The PL is initially dominated by a fast component with a decay time of 1.3±0.1 ns. The PL also contains a second component (about 10%) with a longer decay time (a few ns) and a third even smaller competent that does not decay over the experimental time window (a few ns). The latter presumably arises from localized excitons. We have limited the analysis temporal window to the initial a few ns in the main text when PL from free dark exciton dominates.

Supplementary Note 3: Analysis of the PL and valley dynamics with repetitive pulse excitation

If $I(t)$ is the time-resolved PL intensity under single pulse excitation, the PL dynamics under repetitive excitation with period t_0 can be expressed as $\bar{I}(t) = I(t) + I(t+t_0) + I(t+2t_0) + \cdots$. Here we have assumed that the decay dynamics is unchanged for different excitation pulses. In the case of three-component analysis, we describe the PL intensity as the sum of three independent exponential functions

$$I(t) = \sum_{i=1}^{3} A_i \exp(-t/t_i), \qquad (1)$$

where A_i and t_i (i = 1, 2, 3) denote, respectively, the magnitude and decay time constant of the three components ($t_1 < t_2 < t_3$). Under a repetitive excitation with period t_0, we modify Eq. 1 to be

$$\bar{I}(t) = \sum_{i=1}^{3} A_i \exp(-t/t_i)[1 + \exp(-t_0/t_i) + \exp(-2t_0/t_i) + \cdots]$$
$$= \sum_{i=1}^{3} \bar{A}_i \exp(-t/t_i), \qquad (2)$$

where $\bar{A}_i = A_i\left(1 - \exp(-t_0/t_i)\right)^{-1}$. We fit experimental PL dynamics with Eq. 2, and then reconstruct the PL dynamics for single pulse excitation (Eq. 1). The valley dynamics is calculated as $\rho(t) \approx \frac{I_{K'}(t) - I_K(t)}{I_{K'}(t) + I_K(t)}$, where $I_{K'(K)}(t)$ is the reconstructed PL dynamics.

We show this analysis for the dark hole trion in Supplementary Fig. 10. The three-component model describes well the PL dynamics under different conditions (Supplementary Fig. 10a, b, d, e). There is negligible decay of the valley polarization for the first 3.5 ns, in which the signal-to-noise ratio is still high (Supplementary Fig. 10c, f). We are thus confident that the valley lifetime exceeds several ns as stated in the main text.

Supplementary Note 4: Alternative method for measuring the valley dynamics

In the Fig. 4 of the main text, we have analyzed the valley dynamics of the dark hole trion by comparing the time-resolved PL at the energy of the K and K’ valleys for a given circularly polarized excitation. Alternatively, we can analyze the valley dynamics by comparing the time-resolved PL at a given valley under the RCP and LCP excitations. These two analysis methods,

under most circumstances, are equivalent. Both have been widely applied in measuring the spin lifetimes in the literature for GaAs quantum wells[4–7]. An example is shown in Supplementary Fig. 11, where the valley lifetime of the dark hole trion is determined using the second method. A similar valley lifetime is obtained as in the main text (using the first method). In practice, the second method is more advantageous, since it only involves the rotation of a waveplate to switch between the LCP and RCP excitation, instead of a monochromater to detect the lower- or higher-energy Zeeman-split states.

Supplementary Note 5: Valley dynamics of the dark electron trion

Supplementary Figure 12a shows the time-resolved PL at the lower-energy Zeeman-split state of the dark electron trion for the LCP and RCP excitation. We limit the time window to the first 1.5 ns, in which the signal-to-noise ratio of each PL measurement is at least 5. Supplementary Fig. 12b shows the degree of valley polarization evaluated using the second method discussed above in Supplementary Note 4. A single exponential fit yields a valley lifetime for the dark electron trion to be 3.5$\pm$0.5 ns. To guide the eye, in Supplementary Fig. 12 we have also included a three-component fit function for the PL dynamics and the valley dynamics evaluated from the fit functions.

Supplementary Figures

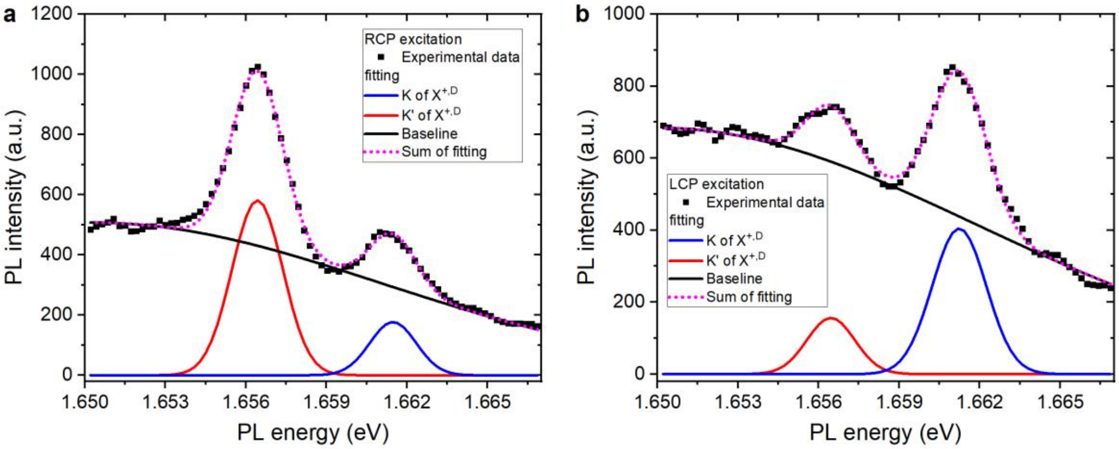

Supplementary Figure 1 | Analysis of the PL spectrum under a magnetic field (hole-doped sample). A hole-doped WSe_2 sample (both gates at – 2.2 V) is excited by the RCP (**a**) and LCP (**b**) excitation under 7.8 T. Each PL spectrum (black symbols) is decomposed into two Gaussians (blue and red curves) and a background (black line, a Gaussian peak with peak position away from $X^{+,D}$). The dotted magenta curves are the sum of all contributions. The valley contrast, estimated from the integrated emission of the two peaks by $\frac{I_{K'}-I_K}{I_{K'}+I_K}$, is 0.53$\pm$0.05 for **a** and -0.44$\pm$0.06 for **b**. The errors represent fitting errors.

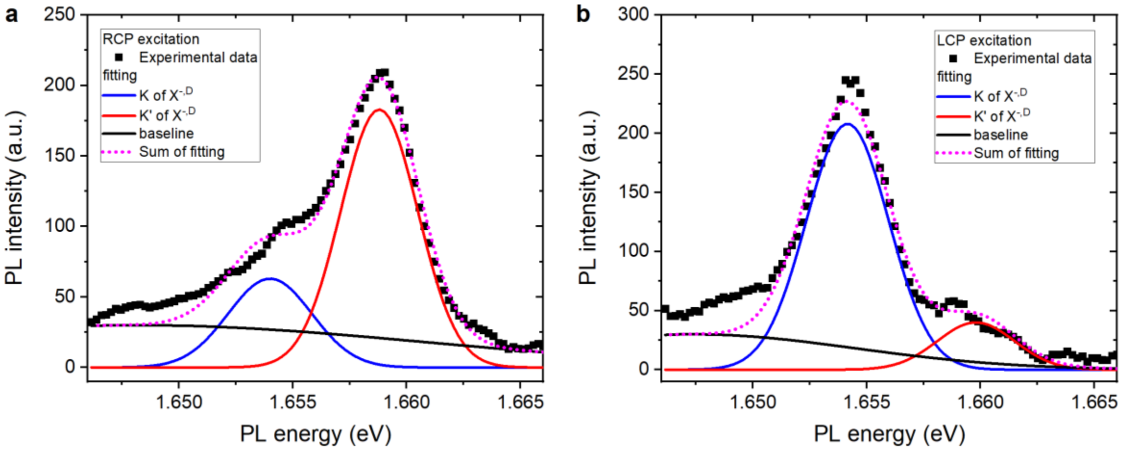

Supplementary Figure 2 | Analysis of the PL spectrum under a magnetic field (electron-doped sample). Same as Supplementary Fig. 1 but for an electron-doped WSe_2 sample (both gates at - 0.5 V). The valley contrast is estimated to be 0.49 $\pm$ 0.09 for **a** and - 0.68 $\pm$ 0.12 for **b**.

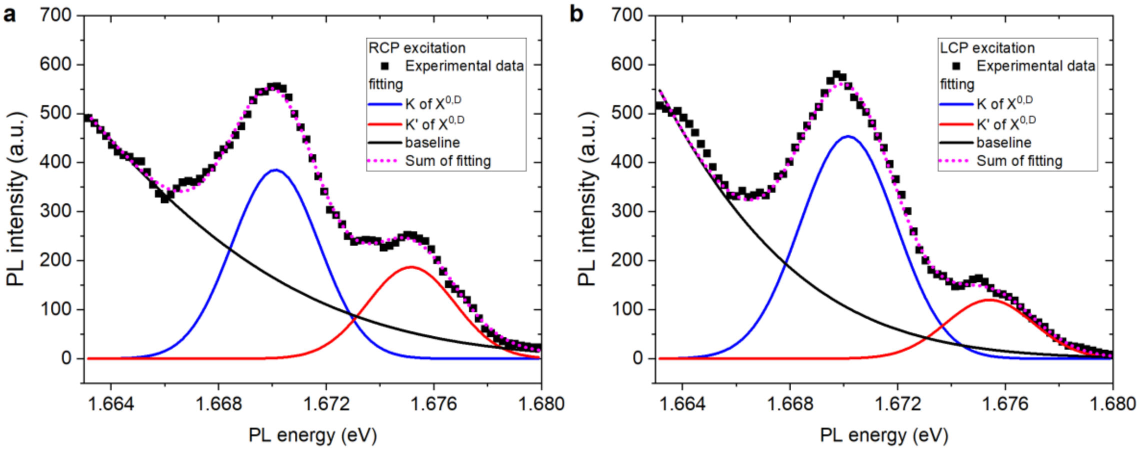

Supplementary Figure 3 | Analysis of the PL spectrum under a magnetic field (neutral sample). Similar to Supplementary Fig. 1 but for a neutral WSe_2 sample (both gates at – 1.75 V). The valley contrast is estimated to be - 0.35 $\pm$0.05 for **a** and -0.58 $\pm$0.08 for **b**.

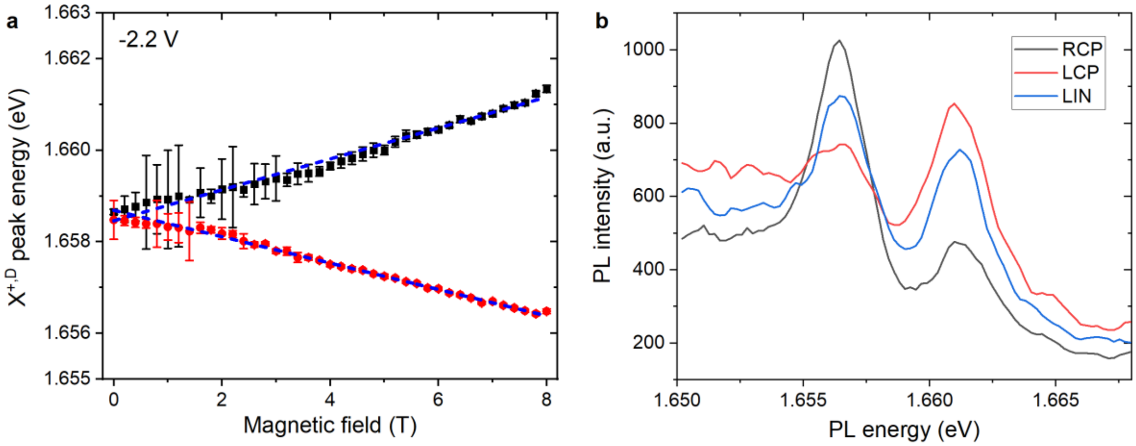

Supplementary Figure 4 | Zeeman splitting of dark hole trion $X^{+,D}$. **a,** The symbols represent the peak energies of the Zeeman-split dark hole trion as a function of OP magnetic field. The energies were determined by fitting the PL spectrum using a combination of two Gaussian functions and a smooth background as shown in Supplementary Fig. 1. The error bars are the fitting uncertainty. The dashed lines are linear fits, corresponding to a g-factor of 10.9 ± 0.1. **b**, PL spectra of the OP channel under a magnetic field of 7.8 T with RCP (black line), LCP (red line) and linearly polarized excitation (blue line).

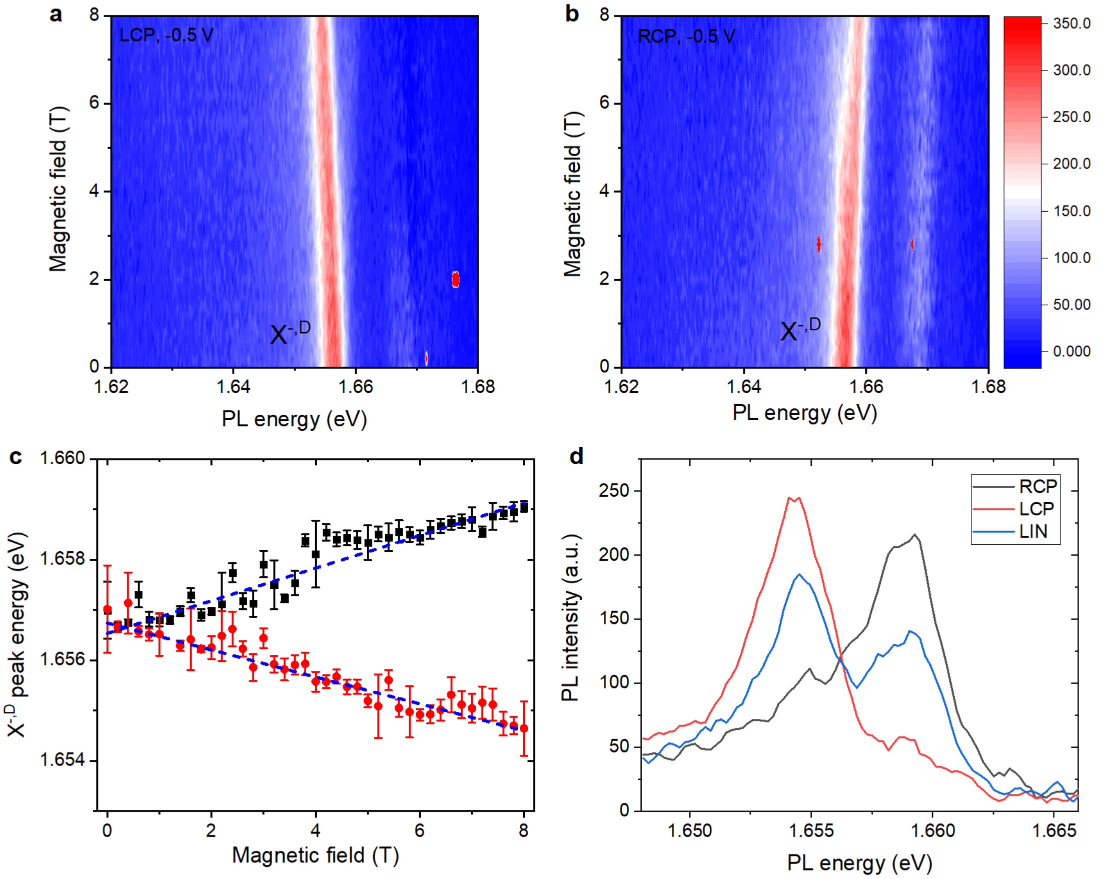

Supplementary Figure 5 | Zeeman splitting of the dark electron trion $X^{-,D}$. **a**, **b**, Contour plot of the PL spectrum of the OP channel as a function of magnetic field for an electron-doped WSe_2 sample (both gates at - 0.5 V). **a** is for the LCP excitation and **b** is for the RCP excitation. **c**, The symbols represent the peak energies of the Zeeman-split dark electron trion as a function of OP magnetic field. Similar to Supplementary Fig. 4, the linear fit reveals a g-factor of 10.2 $\pm$ 0.3 (blue dashed lines). **d**, PL spectra of the OP channel under a magnetic field of 8 T with RCP (black), LCP (red) and linearly polarized excitation (blue line).

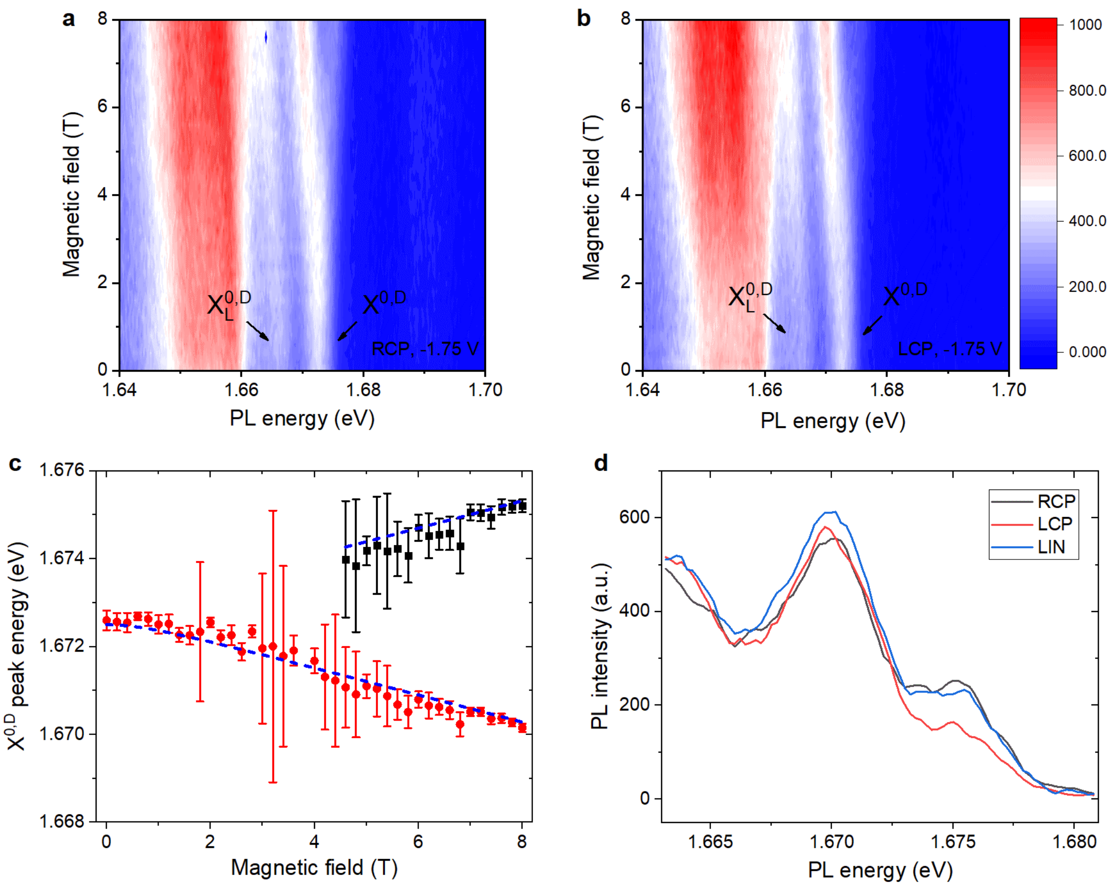

Supplementary Figure 6 | Zeeman splitting of the dark exciton $X^{0,D}$. a, **b**, Contour plot of the PL spectrum of the OP channel as a function of magnetic field for an electron-doped WSe_2 sample (both gates at – 1.75 V). **a** is for the RCP excitation and **b** is for the LCP excitation. The Zeeman splitting (ΔE) in **c** is described by $\Delta E = \sqrt{\delta^2 + (g\mu_B B)^2}$, where δ is the energy splitting at zero magnetic field and fixed at 0.6 meV (obtained from C. Robert et al.[2]). The g-factor of neutral dark exciton is estimated to be 10.8 ± 0.5. **d**, PL spectra of the OP channel under a magnetic field of 8 T with RCP (black), LCP (red) and linearly polarized excitation (blue line).

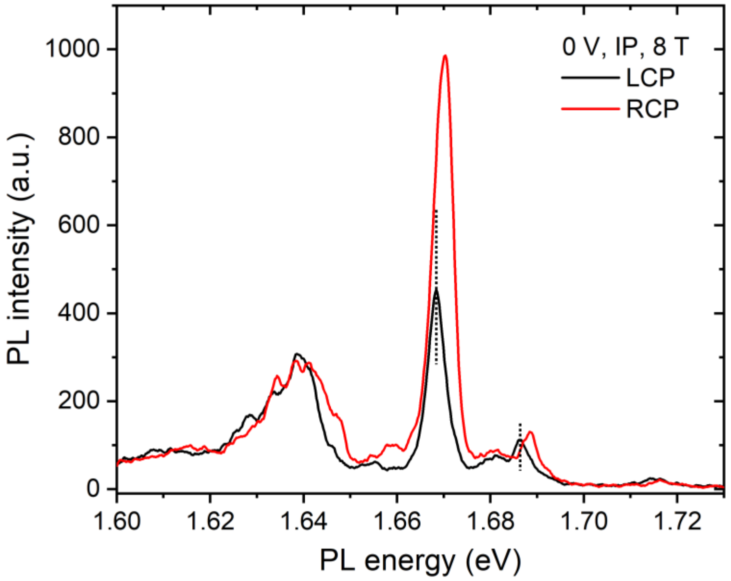

Supplementary Figure 7 | PL spectra of the IP channel under a magnetic field. The PL spectrum of the IP channel for an electron-doped WSe_2 sample (both gates at 0 V) by the LCP (black) and RCP (red) excitation under 8 T. The dotted vertical lines are guides to the eye for the bright electron trion emission peaks under the LCP excitation.

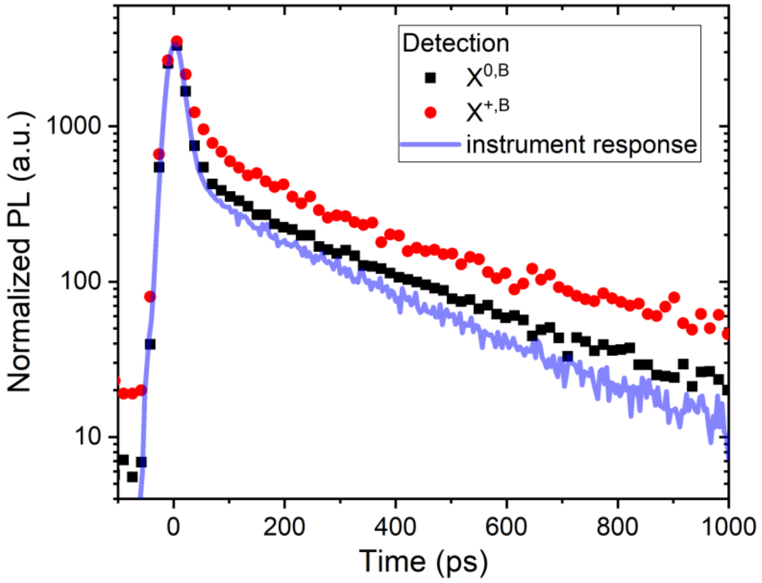

Supplementary Figure 8 | PL dynamics of the bright excitons. Raw data of the time-resolved PL of the bright neutral exciton (black symbols) and hole trion (red symbols) of a hole-doped WSe_2 sample (gate voltages at – 2.2 V)**.** The blue curve is the instrument response function obtained by measuring a fs laser pulse. All the curves have been normalized to have the same peak value.

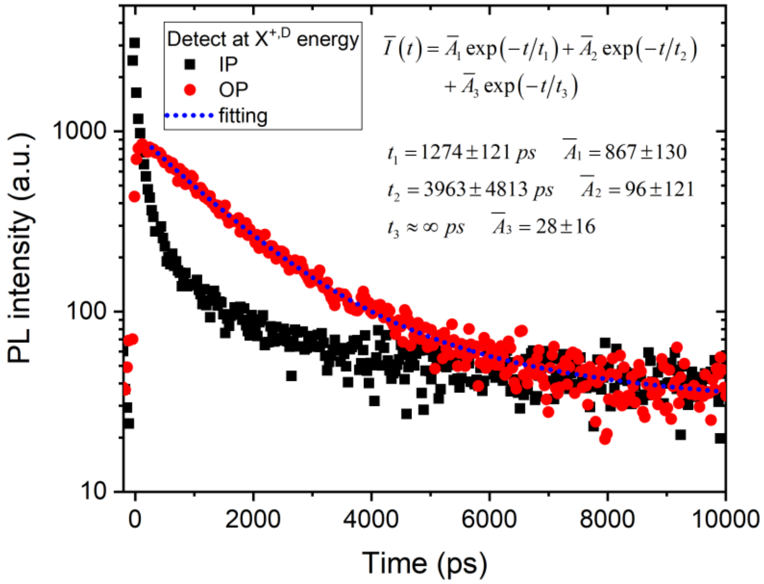

Supplementary Figure 9 | PL dynamics of the dark hole trion. Time-resolved PL at the energy of the dark hole trion in the OP (red symbols) and IP (black symbols) channel of a hole-doped WSe_2 sample (gate voltages at – 2.2 V). The data have been deconvoluted with the IRF shown in Supplementary Fig. 8. The blue dotted curve is a three-exponential fit (inset) to the OP channel result, revealing a recombination time constant of 1.27 ± 0.12 ns for the fastest exponential decay component, which is likely related with the free dark hole trions.

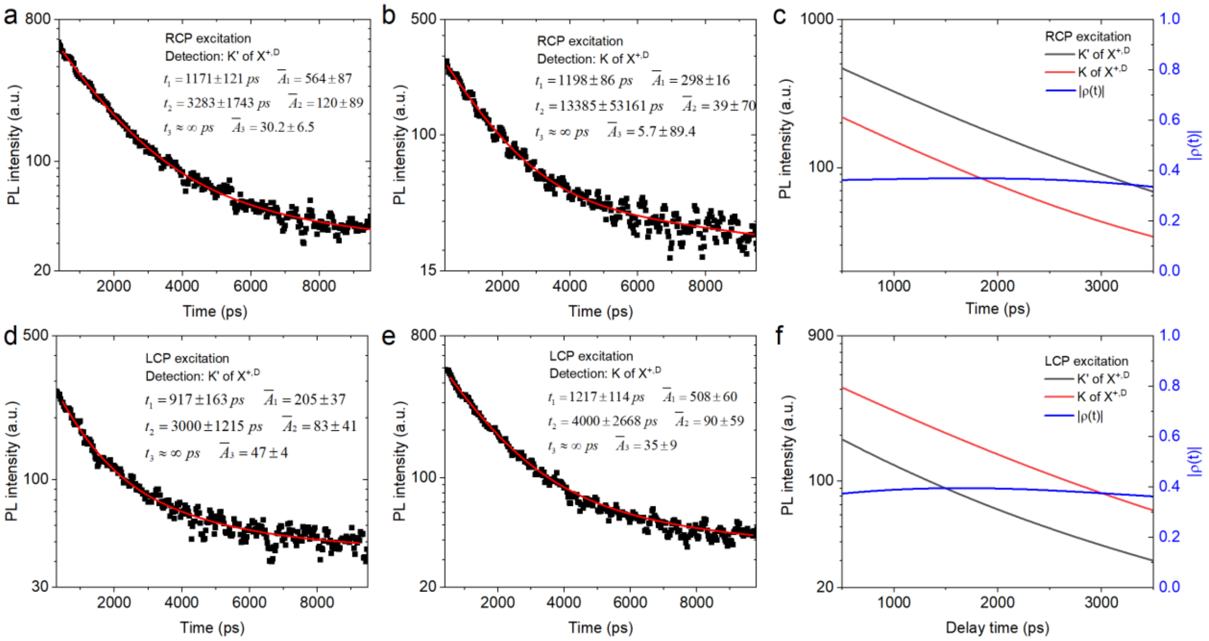

Supplementary Figure 10 | Three-exponential fit of the PL dynamics of the dark hole trion. **a, b, d, e,** Black symbols are experimental data. Red curves are fit as described in the Supplementary Note 3 for the PL dynamics from the K' and K valley state of $X^{+,D}$ with RCP (**a**,**b**) and LCP (**d**,**e**) excitation. **c, f,** Black and red curves are the reconstructed PL dynamics under single excitation from the K' and K valley state of $X^{+,D}$, respectively. Blue curves are the valley contrast $\rho(t)$ defined as $\frac{I_{\mathrm{RCP}}-I_{\mathrm{LCP}}}{I_{\mathrm{RCP}}+I_{\mathrm{LCP}}}$, calculated from the reconstructed PL dynamics. Negligible changes are observed within the time window of the first 3.5 ns.

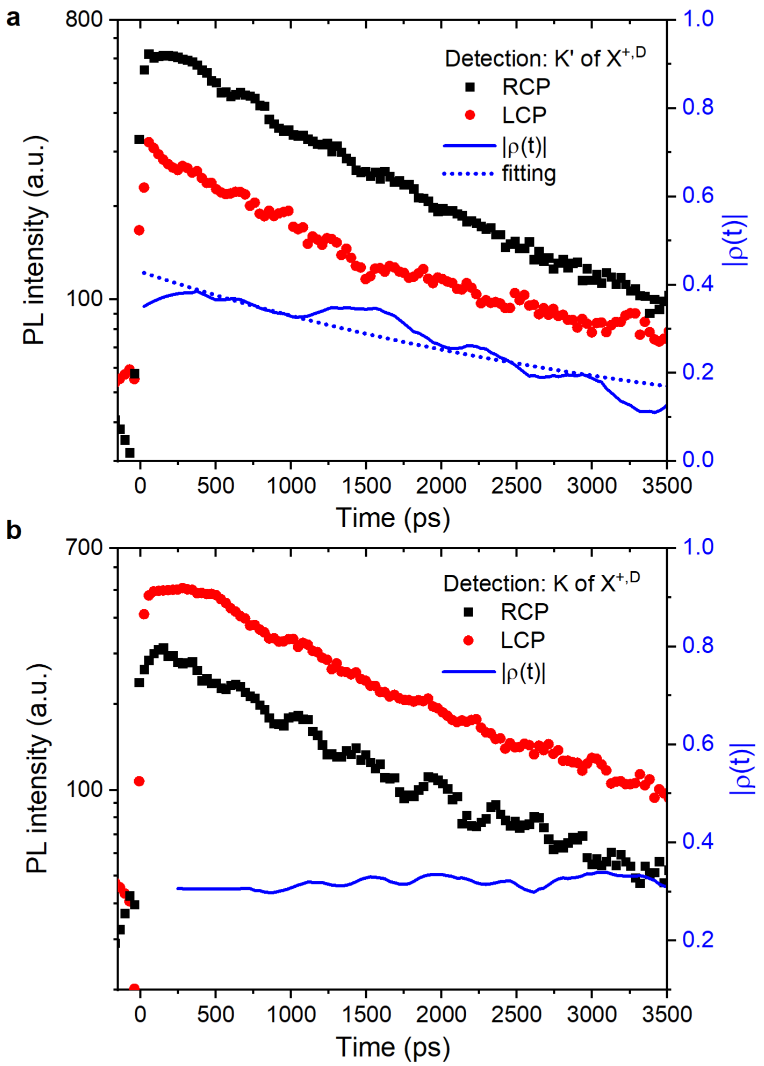

Supplementary Figure 11 | PL dynamics of the dark hole trion under 8 T (an alternative method). The time-resolved PL of a hole-doped WSe_2 sample (both gates at – 2.2 V) is monitored at the K' (**a**) and K valley (**b**) for the dark hole trion. The black and red symbols represent the PL under the RCP and LCP excitations, respectively. The solid blue curves represent the valley contrast $\rho(t)$ defined as $\frac{I_{\mathrm{RCP}}-I_{\mathrm{LCP}}}{I_{\mathrm{RCP}}+I_{\mathrm{LCP}}}$, where $I_{\mathrm{RCP(LCP)}}$ is the PL intensity with the RCP(LCP) excitation. The dotted blue curve in **a** represents a single-exponential fit to the dynamics of $\rho(t)$ for the first 3.5 ns, corresponding to a decay time constant of 3.8 ± 0.2 ns. No meaningful fit for the dynamics of $\rho(t)$ in **b** can be obtained, as there is negligible decay.

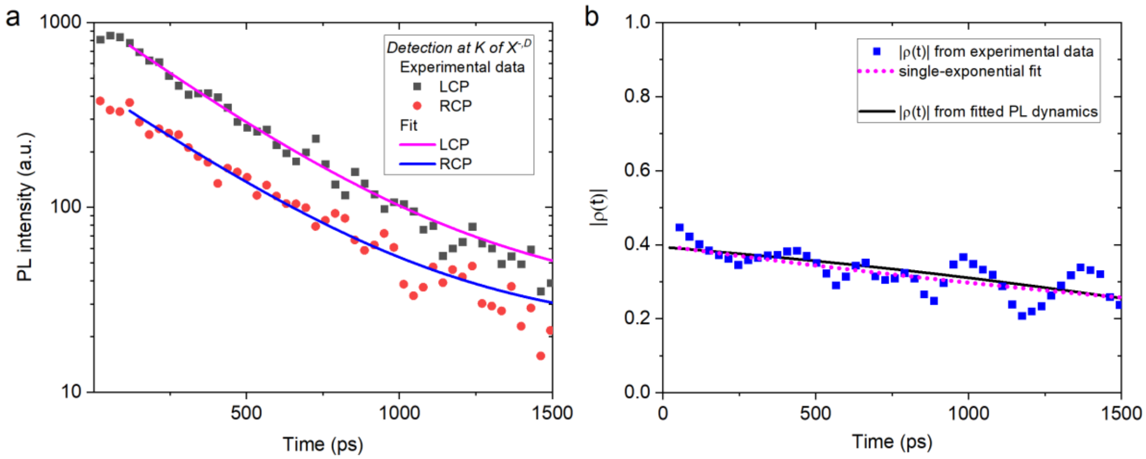

Supplementary Figure 12 | Valley dynamics of the dark electron trion. **a**, The time-resolved PL monitored at the K valley of the dark electron trion (the lower-energy peak of the Zeeman-split states) under a magnetic field of 8 T of an electron-doped WSe_2 sample (both gates at - 0.5 V). The black and red symbols represent the PL with the RCP and LCP excitation, respectively. The solid magenta and blue curves respectively represent three-exponential fit to the experimental data with LCP and RCP excitation. **b**, The valley contrast $\rho(t)$ defined as $\frac{I_{\mathrm{RCP}}-I_{\mathrm{LCP}}}{I_{\mathrm{RCP}}+I_{\mathrm{LCP}}}$, calculated using the experimental data (symbols) and the fit functions (solid line) under LCP and RCP excitations in **a**. The dotted curve is a single-exponential fit to the symbols , revealing a decay time constant of 3.5 $\pm$ 0.5 ns.

Supplementary References

1. Barbone, M. *et al.* Charge-tuneable biexciton complexes in monolayer WSe_2. *Nat. Commun.* **9**, 3721 (2018).
2. Robert, C. *et al.* Fine structure and lifetime of dark excitons in transition metal dichalcogenide monolayers. *Phys. Rev. B* **96**, 155423 (2017).
3. Li, Z. *et al.* Revealing the biexciton and trion-exciton complexes in BN encapsulated WSe_2. *Nat. Commun.* **9**, 3719 (2018).
4. Damen, T. C., Leo, K., Shah, J. & Cunningham, J. E. Spin relaxation and thermalization of excitons in GaAs quantum wells. *Appl. Phys. Lett.* **58**, 1902–1904 (1991).
5. Muñoz, L., Pérez, E., Viña, L. & Ploog, K. Spin relaxation in intrinsic GaAs quantum wells: Influence of excitonic localization. *Phys. Rev. B* **51**, 4247–4257 (1995).
6. Roussignol, P. *et al.* Hole polarization and slow hole-spin relaxation in an n-doped quantum-well structure. *Phys. Rev. B* **46**, 7292–7295 (1992).
7. Vinattieri, A. *et al.* Exciton dynamics in GaAs quantum wells under resonant excitation. *Phys. Rev. B* **50**, 10868–10879 (1994).
